# Designing an Integrated Care Initiative for Vulnerable Families: Operationalisation of Realist Causal and Programme Theory, Sydney Australia

**DOI:** 10.5334/ijic.3980

**Published:** 2019-07-25

**Authors:** John G. Eastwood, Miranda Shaw, Pankaj Garg, Denise E. De Souza, Ingrid Tyler, Lauren Dean, Morag MacSween, Michael Moore

**Affiliations:** 1 School of Women’s and Children’s Health, The University of New South Wales, Sydney, NSW, AU; 2 Ingham Institute of Applied Medical Research, Liverpool, NSW, AU; 3 Charles Perkins Centre, Menzies Centre for Health Policy, Discipline of Child and Adolescent Health, and School of Public Health, University of Sydney, Sydney, NSW, AU; 4 Sydney Institute for Women, Children and their Families, Camperdown, NSW, AU; 5 Community Health Services, Sydney Local Health District, Camperdown, NSW, AU; 6 School of Humanities, Nanyang Technological University, SG; 7 Dana Lana School of Public Health, University of Toronto, ON, CA; 8 Fraser Health Authority, Surrey, BC, CA; 9 Department of Family and Community Services, Sydney, NSW, AU; 10 Central and Eastern Sydney PHN, Ashfield, NSW, AU

**Keywords:** Child and Family, Integrated Care, Critical Realism, Design

## Abstract

**Introduction:**

In July 2015 Sydney Local Health District (SLHD) implemented an integrated care initiative for vulnerable families in the Inner West region of Sydney, Australia. The initiative was designed as a cross-agency care coordination network that would ensure that vulnerable families: had their complex health and social needs met; kept themselves and their children safe; and were connected to society. We will describe the development of the design that drew on earlier realist causal and program theoretical work.

**Methods:**

Realist causal and program theory were used to inform the collaborative design of an initiative for vulnerable families. The collaborative design process included: identification of desirable and undesirable outcomes and contextual factors, stakeholder consultation, interagency planning, and development of a service proposal.

**Results:**

The design elements included: identification of vulnerable family cohorts; care coordination; evidence-informed intervention(s); general practice engagement and support; family health improvement; placed-based neighbourhood initiatives; interagency system change and collaborative planning; monitoring of individual and family outcomes; and evaluation.

**Conclusions:**

The design study described advances toward the implementation of a whole-of-government integrated health and social care initiative. The initiative was designed as a cross-agency care coordination network that would ensure that vulnerable families: had their complex health and social needs met; kept themselves and their children safe; and were connected to society. In so doing we aim to break intergenerational cycles of poverty, violence and crime, poor education and employment opportunities, psychopathology, and poor lifestyle and health behaviours, through strengthening family resilience, improving access to services, and addressing the social determinants of health and wellbeing.

## Introduction

We have previously argued that chronic and complex health conditions have their origins in early childhood and that there is complex intergenerational transmission of their antecedents through probable genetic, epigenetic, behavioural, psychological and psychosocial mechanisms [1]. Our empirical and theory building studies had identified the important causal role that maternal stress plays in those developmental origins of health and disease. In addition, the critical realist theory proposed that social policy and social services play an important role in buffering the adverse effects of isolation and lost expectations [1]. The findings from those earlier studies have been used to inform the development of interagency integrated care initiatives for children and their families described here.

In July 2015 Sydney Local Health District (SLHD) implemented an integrated care initiative for vulnerable families in the inner west region of Sydney, Australia. The initiative was designed as a cross-agency care coordination network that would ensure that vulnerable families: had their complex health and social needs met; kept themselves and their children safe; and were connected to society. The initiative was part of a New South Wales (NSW) Government Integrated Care Strategy initiated in March 2014 and drew on the analysis of an earlier design study for vulnerable families undertaken from 2010 to 2014 [2].

The objective of the NSW Integrated Care Strategy was to transform the delivery of care to improve health [and wellbeing] outcomes for patients and reduce costs deriving from inappropriate and fragmented care, across hospital and primary care services. The NSW Government indicated that this objective was to be achieved by: a) focusing on organising care to meet the needs of targeted patients and their carers, rather than organising services around provider structures; b) designing better connected models of health [and social] care to leverage available service providers to meet the needs of our smaller rural communities; c) improving the flow of information between hospitals, specialists, community and primary care providers; d) developing new ways of working across State government agencies and with Commonwealth funded programs to deliver better outcomes for identified communities; and e) providing greater access to out-of-hospital community-based care, to ensure patients receive care in the right place for them.

The NSW Government’s stated policy intent was to respond to the challenges of an ageing population and increased numbers of people living with chronic and complex conditions by investing in new, innovative models of integrated care, transforming the health system to routinely deliver person-centered, seamless, efficient and effective care, particularly for people with complex, long term conditions. Funding was made available, through a competitive grant process, for health districts to develop innovative integrated care projects. The functional elements of the NSW Health Integrated Care Strategy are shown at Table 1.

**Table 1:** NSW Ministry of Health Integrated Care Functional Components.

Functional Component	Key Feature

Patient and carer empowerment	

**Engaging the patient/carer in care planning**	The implementation of processes and systems that ensure the integrated care plan meets the needs and preferences of patient/carers as defined by patients or carers themselves (shared decision making).
**Using patient reported measures in care delivery**	The implementation of a system of patient reported measures for enrolled patients that measure both the patient’s perceptions of both their care experience and their outcomes, due to the care that they receive. This includes the timely provision of the information to clinicians/team delivering care to enable shared care planning/shared decision making.
**Supporting and promoting self-management**	A set of defined care interventions specific to the targeted patient cohort to support self-management. This also includes strategies to increase capacity for patients and carers to better self-manage their condition.
**Building patient/carer health literacy**	The implementation of processes and systems (such as training and information) that improve the patient’s understanding of their health condition(s), how to maximise their ability to manage it themselves, how/when to access health services and what role they play in managing their health condition(s). This also includes care plan access, and active participation to the extent possible in care planning.
**Patient identification and selection**	

**Defining local health needs**	The set of local health system parameters which broadly identify the types of patients that require the implementation of an integrated care pathway to improve the effectiveness of healthcare delivery (such as potentially avoidable hospital admission, ED presentations, delays in receiving specialist treatment).
**Identifying target cohorts**	Patient level parameters (such as demographic, e.g. age; clinical, e.g. diagnosis; utilisation, e.g. number of medications; other, e.g. measure of social disadvantage) that define the group of patients that will be targeted/enrolled in the integrated care program.
**Developing systematic approaches to risk identification**	The standardised approach to risk identification (such as signs of health deterioration) and methodology (such as automated processes in PAS/EMR/EHR) for identification of the targeted cohort of patients who would benefit from an integrated model of care. The targeted risks and cohorts can vary locally, and can vary over time within locality as programs mature.
**Innovative ways of working**	

**Establishing new business models**	The identification and implementation of business models across the continuum of care are being to promote care delivery which improves patient care and experience through improved coordination and integration. The models sit alongside service models (which operationalize service delivery). They potentially incorporate financial and/or non-financial elements. The models may include the selection of alliance partners (such as GPs, NGOs or other government organisations) and investment in new roles, as well as the use of known business models (such as Person Centred Medical Homes or a Commissioning Framework).
**Ensuring appropriate and timely access to specialist care**	Needs for the identified cohort. The function may be achieved in a number of different ways (for example, quarantining appointments in hospital based clinics or purchasing services from a telehealth provider).
**Shared/joint care planning and management with the patient/carer**	The development of shared or joint care planning and care management strategies between the initiator of the care plan, the patient, and other healthcare professionals who are to be involved in the care and service delivery to targeted patients.
**Establishing roles focused on organising patient-centred care**	The establishment of roles (such as case managers, care navigators, care facilitators) to support the implementation of the integrated care model of care across care settings (such as hospital, primary care, specialist care, community care).
**Embedding agreed models of care**	The uptake of models of care for patients with specified conditions that are based on evidence based medicine and adhered to by those clinicians seeing targeted patients. This includes the process of designing and agreeing the models with stakeholders to optimise uptake.
**Primary and Community care as the hub**	

**Connecting people to their healthcare team**	The assignment of targeted patients to a clinical provider (individual/practice) whose role is to be the lead clinical provider with responsibility for the shared care plan and initiating communication with other care providers (such as specialist, GP, aged care, community care).
**Systematic assessment, review of patients**	The implementation of a system of standardised assessments, regular patient reviews, and uploading of relevant clinical metrics by clinical care providers based on developed integrated care pathway protocols.
**Building capacity/capability in primary and community care**	The enhancement of resources (such as care navigators, training programs, care pathways, share care planning tools) in the primary and community care settings to support integrated care delivery to targeted patients.
**Information Sharing**	

**Establishing a trackable cohort list**	The establishment of an electronic patient list/register that identifies all patients enrolled in the integrated care initiative and enables the monitoring of the patient journey, as reflected through the patient’s use of healthcare services.
**Establishing shared access to patient information**	The extent of electronic patient information on enrolled patients available to clinicians across care settings who are delivering the agreed integrated model of care (such as care plans, e-referral, discharge summaries, medication profiles, test results, service events).

The Inner West Sydney District collaborated to design initiatives for vulnerable families in 2013. A critical realist design method was applied and included programme theory developed from an analysis of our realist causal propositions [3]. The resulting Theory of Change (ToC) Logic Model included three design components, namely: sustained health home visiting; family and community integrated service development; and infrastructure support [3]. The three design elements were further developed for inclusion in: 1) a Working with Vulnerable Families Business Case, and 2) Child and Family Health Planning Priorities (Table 2). Importantly the design included population, system and individual-level health and social care elements.

**Table 2:** Design elements of previous planning.

Design Component	Business Case	Child and Family Health Planning Priorities

Sustained Health Home Visiting (SHHV)	Antenatal screening and risk stratification Perinatal pathways and coordination Sustained home visiting commencing before birth Second tier allied health and medical services, pathways and coordination Universal maternal, child and family services with proportionate support according to need	Review and strengthen perinatal coordination Strengthen Aboriginal program (Yana Muru) New SHHV in Canterbury LGA focusing on CALD families Enhance SHHV in Sydney LGA focusing on Redfern and Waterloo suburbs Strengthen Tier 2 support services including access pathways
Family and Community Integrated Service Development (FCISD)	Integrated service models including wrap-around and family group conference model Targeted parenting programmes Domestic violence intervention High risk infant tracking models “Hub” and “place-based” community building and service coordination Universal family and community capacity building (health and wellbeing promotion)	Interagency collaborative planning Development of interagency models of care for “high need” schools and early childhood centres Commence neighbourhood “hub” development in Redfern social housing estate Enhanced collaborative interagency parenting communication strategy (phone app and web development)
Infrastructure Support (IS)	Child and family public health (epidemiology, programming, research and evaluation) System change strategies Service capacity building Project Management and leadership	Child and family epidemiology Evidence-informed programming Evaluation of perinatal referral pathways Study of universal well child care system Web-based health pathway development Development of well child care and psychological trauma workforce training packages Leadership and technical support to interagency planning groups

**Table 3:** Integrated Care Programme Design Elements.

	Design Component	Inner West Sydney Collaborative Design	Ministry of Health Integrated Care Policy	Design Elements

1	Shared identification and intake	Strengthen existing perinatal screening and coordination system through review, training and monitoring High risk infant tracking models	Identifying target cohorts Developing systematic approaches to risk identification Establishing a trackable cohort list Establishing shared access to patient information	Shared identification Shared risk stratification Pathways to care Shared intake systems
2	Care Coordination	Strengthen existing perinatal screening and coordination system through review, training and monitoring Strengthen Tier 2 support services Integrated service models including wrap-around and family group conference model High-risk infant tracking models	Engaging the patient/carer in care planning Supporting and promoting self-management Using patient reported measures in care delivery Ensuring appropriate and timely access to specialist care Shared/joint care planning and management with the patient/carer Systematic assessment, review of patients Connecting people to their healthcare team	Patient centered care Strength-based care coordination Facilitated access to specialist care Shared care planning Shared assessment and review of patients Wrap around connecting people to health and social care team
3	Evidence informed practice	Strengthen current SHHV by training, resourcing, management support Integrated service models including wrap-around and family group conference model Targeted parenting programmes		Sustained Health Home Visiting Wrap-around service model Family Group Conferencing Targeted Parenting Programmes
4	General Practice engagement and support		Connecting people to their healthcare team Systematic assessment, review of patients Building capacity/capability in primary and community care	Connecting families to general practice “health home” Supporting general practice to engage and support families Capacity building of general practice
5	Family Health Improvement	Review and strengthen universal services Targeted parenting programmes Universal family and community capacity building	Building patient/carer health literacy	Universal family health literacy Parent education and support programmes Sector-wide capacity building
6	Place-based initiatives	Implement new tiered model of SHHV in Canterbury, Redfern and Waterloo Integrated service models including wrap-around and family group conference model “Hub” and “place-based” community building and service coordination	Engaging the patient/carer in care planning Defining local health needs Connecting people to their healthcare team Building capacity/capability in primary and community care Establishing shared access to patient information	Place-based initiatives in City of Sydney and City of Canterbury/Bankstown Integrated care pilot projects to include: local needs analysis, consumer consultation, “service hub”, wrap-around service provision, family group conferencing, community building and service coordination
7	System Change	Strengthen existing perinatal screening and coordination system through review, training and monitoring Review and strengthen existing perinatal screening and coordination system project management and leadership Sector capacity building projects System change projects	Establishing new business models Establishing roles focused on organising patient-centred care Embedding agreed models of care Defining local health needs	New business models Strengthen existing perinatal screening and coordination system Shared outcomes, assessment tools, models of care, and evaluation Sector capacity building projects System change projects
8	Child and family Outcomes	Child and Family public health (research, programme, evaluation)	Using patient reported measures in care delivery	Patient reported measures
9	Evaluation	Child and Family public health (research, programme, evaluation)	Defining local health needs	Critical realist evaluation Population outcome evaluation

During 2014 the Inner West Sydney District collaborative planning for child and family health and wellbeing was extended to focus on improved outcomes for all children and their families. At the time of the launch of the NSW Integrated Care Strategy in March 2014, the planning process had identified four strategic themes, namely: improving system capacity; health and wellbeing promotion; early intervention and supporting place-based initiatives. In addition a detailed outcome framework had been developed based on earlier studies of child and family population-level indicators [4,5].

This paper will describe the design development for an integrated care initiative for vulnerable families including children. The design will draw on our earlier causal and programme theoretical work, the 2013 collaborative design for vulnerable families, and the 2014 NSW Government policy framework for integrated care [2,3,6]. The research is part of two ongoing programmes of research and programme development that seeks to 1) build and confirm a “Theory of Neighbourhood Context, Stress, Depression, and the Developmental Origins of Health and Disease (DOHaD)” [7]; and 2) strengthen the delivery of well child care through mixed method theory building and the application of interagency policy and program interventions. The work was undertaken during 2014 and 2015 with the integrated care initiative commencing July 2015.

## Theory and Methods

As previously described [6,8,9], the overall research design is a longitudinal, multilevel, critical realist design and evaluation of applied programme interventions that seek to break intergeneration cycles of social disadvantage and poor child health and development outcomes and strengthen delivery of well child care. Intervention initiatives were designed and implemented by interagency and community collaborations. In doing so we aimed to move from “explaining underlying social mechanisms to generate social interventions in partnership with the affected populations” [10].

**Figure 1:**
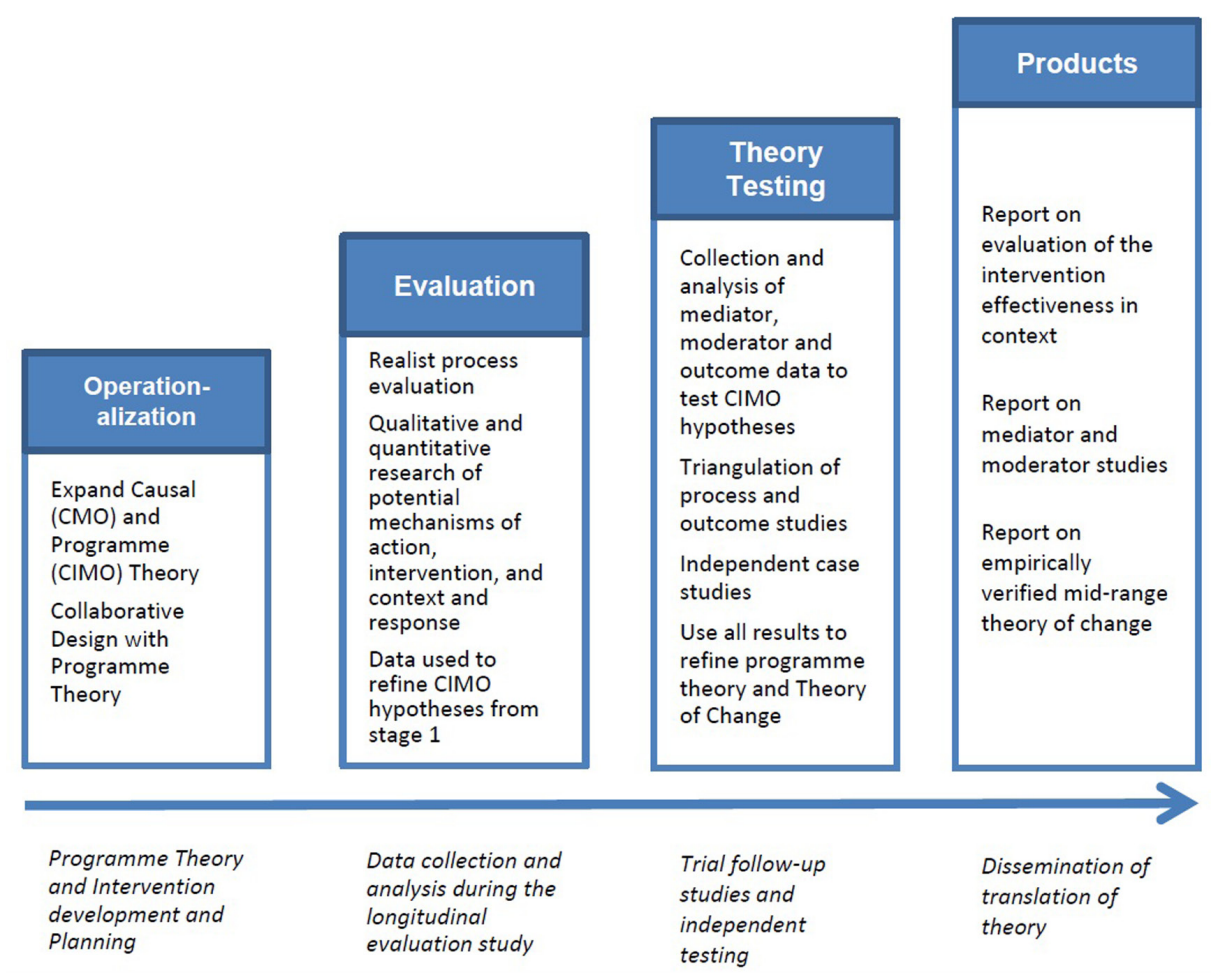
Summary of Research Programme.

The main research programme will consist of four phases (Figure 1). The methodology used for the four phases is reported separately [9]. In summary the four phases are: 1) operationalisation of programme theory and intervention development and planning; 2) evaluation of the interventions; 3) theory testing studies; and 4) dissemination of the findings. In this paper we report on one of the collaborative design projects undertaken in Phase 1: Operationalisation. The operationalisation of causal and programme theory is briefly described in the Results section with Table 4. The full analysis will be reported separately.

**Table 4:** CIMO Propositions.

Theorised Contextual Conditions (Figure 2) [C]	Present contextual mechanisms activated [C_M_]	Proposed Intervention Design Elements (Table 1) [I]	Postulated Intervention Programme Mechanisms (Table 1) [M_P_]	Postulated psychological, motivational and behavioural Outcomes [O]

Self – Self-identity and individual’s experience				

Lack of partner and family support, Distrust of services, Limited treatment access	Stress mechanism activated causing anxiety and depression	Friendship and family support, Professional support, Medication, Treatment	Activate mediating mechanisms of family, peer and professional support to strengthen and build trusting relationships with peers, family and clinicians through SHHV and FCISD Design Components.	Decreased depression and anxiety
Lifetime trauma, Loss, Being alone, Isolation	Stress mechanism activated arising from mismatched expectations, and loneliness	Family and peer support, Home visiting, Telephone support		Increased perceived support
**Situated Activity – Face to Face activity**				

Services unavailable or poor access, Services not trusted, Services not skilled	Absence of trusted professional support mechanism	“wrap-around” services, Family Conferences, Workforce training	Activate services mechanisms that are client, peer and neighbourhood focused, and trauma and evidence informed through FCISD and IS Design Components.	Improved perceived access to skilled and trusted services
Community distrust, Low social capital and cohesion, crime, unemployment	Absence of trusted neighbourhood and community support mechanism	“wrap-around” services, Family Conferences, Public health, Social work services		Improved perceived support from neighbours and community
**Intermediate Level social and service organisation**				

Unhelpful intake and referral practices, Lack of service, knowledge and trust	Absence of specialist service support mechanism for front-line professionals	Strengthened pathways and design Collocation of services	Activate mechanisms related to trust and confidence with service network, increased local social capital, community trust and community safety Activate mechanisms relating to improved coordination and access to services and information through FCISD and IS Design Components.	Improved perceived access to services that are “wrapped” around front-line workers
Weak social networks, community trust, community safety, available social services, access to information	Social level stress mechanisms relating to class, position, racism, segregation, crime and neighbourhood decay are activated tending to increase psychological stress	Population and community level interventions in neighbourhoods and communities		Decrease in psychological stress of individuals and families
**Macro Level social and service organisation**				

Migration, Mega-malls pull service activity away from neighbourhoods, Urban development	Activation of social level stress mechanisms tend to hinder the activation of social level buffer mechanisms	Population and community level interventions in neighbourhoods and communities	Activate mechanisms related to increased social level activities in deprived neighbourhoods. Activate mechanisms related to increased migrant related social activities among ethnic populations through FCISD and IS Design Components.	Increase in perceived social level buffers
Immigration policy, Racism, Media policy, Global market, Settlement patterns, Ethnic bonding networks, Access to services	Migrant related social level mechanisms including acculturation, cultural practices and integration tend to decrease social level stress	Ethnic and cultural specific community and population level interventions		Increase in perceived migrant social level buffers

*Note*: SHHV-Sustained Health Home Visiting; FCISD – Family and Community Integrated Service Development; IS-Infrastructure Support.

### Critical realism and programme design

**Figure 2:**
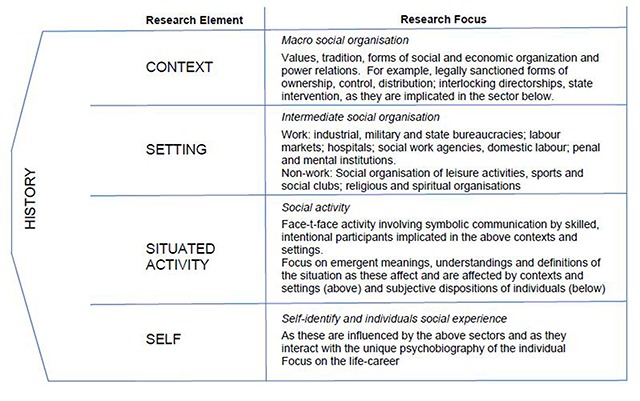
Research Map [11].

As noted above, critical realism provides the philosophical and methodological underpinning to this programme of work. Critical realism seeks to discover the structures (C) and underlying mechanisms (M) that cause empirically observed patterned events or outcomes (O). These events are tendencies that result when certain conditions exist, or remain unrealised if the conditions are absent. Examining the pre-existing structural conditions of a context is therefore important. Critical realism also holds that mechanisms, in natural and social reality, are stratified. Depression (event), in the strata of the self, is governed by physical, biochemical and psychological mechanisms and laws. These mechanisms are not governed by laws operating at the level of social activity, but are nevertheless affected by them. Critical realist theories therefore, may explain event mechanisms by antecedent causes, or explain mechanisms operating at one level by those operating at a more basic level. A higher-level mechanism, is said to be emergent from a more basic mechanism. Layder [11] illustrated this layering of reality in his Research Map (Figure 2). This study uses a modification of Layder’s levels, namely, Self, Situated Activity, Intermediate Level and Macro Level. Mechanisms, emergence, a hierarchy of levels, and pre-existing historical conditions are all central to the critical realist design process described here.

Realist causal propositions are expressed in terms of mechanisms (M), context (C), and outcomes (O). The MCO propositions in our previously reported theory [1] are in the MCO form proposed by Danermark and colleagues [12]. For evaluation studies, Pawson and Tilley [13] have proposed a CMO configuration. In realist programme evaluation terminology the mechanism (M) is an intervention mechanism (IM), and not a causal mechanism. Denyer and colleagues [14] draw attention to the importance of specifying the intervention separate from the mechanism and proposed the use of a CIMO-logic (Context, Intervention Mechanism, Outcome). Thus a CIMO is a hypothesis that the programme theory produces a change (O) because of the action of an intervention (I) on an underlying mechanism (M) operating in particular contexts (C). We will use the CIMO logic in this study and will apply it to the development of the Theory of Change (ToC) logic model (Figure 4).

Realist programme evaluation usually starts with a programme that has been already designed. The approach assumes that whenever a programme is implemented it is testing an existing programme theory consisting of realist programme hypotheses (CMOs). The process of designing a programme intervention using realist causal and programme theory is not well explicated. For the purposes of this study we have drawn on the work of Keller and colleagues [15] who present a realist design-evaluation framework that combines design theory and realist evaluation.

### Collaborative Design

The collaborative design of the integrated care initiative involved: 1) consultation and planning forums; 2) shared outcome planning; 3) collaborative interagency planning; and 4) preparation of a fully funded business plan, ToC and logic model.

The development of a ToC using collective and collaborative processes can be difficult [16]. We used the set of steps proposed by Mackenzie and Blamey:

1. Identification of the long-term outcomes that the initiative seeks to achieve
2. Identification of the interim outcomes and contextual features that will be required to meet these longer-term outcomes
3. Specification of the activities that will be put into place and the contextual requirements to realise these interim outcomes
4. An explicit recognition of the resources that will be required to turn these goals into reality.

The design analysis integrated our earlier causal and programme theoretical work [6], analysis of local child and family population-level indicators [5]; the 2013 collaborative design for vulnerable families [2]; and the 2014 NSW Government policy framework for integrated care. Consequently the critical realist theoretical framework guiding the collaborative effort incorporated 5 elements:

1. Historical analysis of the context to theorise the pre-existing social structures and mechanisms [17]
2. Proposed design elements of an intervention, stemming from inputs from forums, realist syntheses, interviews and collaborations during 2013 and 2014 [2,6]
3. Proposed design elements arising from an analysis of the NSW Government Integrated Care Strategy
4. The development of a programme theory hypothesising the pre-existing situational conditions and causal mechanisms, and specifying how the proposed intervention would trigger desired psychological, motivational and behavioural responses to bring about change [18]
5. The construction of Theory of Change (ToC) logic model explicating a proposed implementation theory [18].

### Ethics

The planning undertaken here did not include human subjects. Ethical approval was not sought. The indicator reports used secondary data and did not require ethics approval. The earlier cited mixed method multilevel studies had ethics approval from the University of New South Wales.

## Results and Design

The proposed design elements for the integrated care initiative drew on: 1) collaborative design processes undertaken for vulnerable families in 2013 and the 2014 planning for a five-year child health and wellbeing plan; and 2) the NSW Ministry of Health Integrated Care Strategy Table 3.

**Shared identification and intake:** A population-based approach was proposed for identifying the most vulnerable families, developing cross-agency assessment and referral pathways, and improving hospital maternity services’ recognition of the needs of families. The Pathways to Care component included: strengthening of perinatal screening and coordination systems; establishment of a centralised intake system; development of primary care referral pathways using the New Zealand Canterbury HealthPathways Sydney tools; development of a shared family risk assessment tool; building of a electronic medical record tool to support clinicians identifying families; and the building of a database to support the monitoring of outcomes among identified family members.

**Care coordination:** The design provided for the establishment of a nurse-led family care coordination service model that would support families over a long time period with the intention of bridging the episodic nature of existing family support services. The proposed role was to: a) provide leadership and support in the building of local service networks and referral pathways for vulnerable families; b) liaise with and support service providers to ensure referral to appropriate services in accordance with shared care plan; c) coordinate and track service provision for the identified vulnerable families, including ongoing information coordination for providers; d) provide information, support and referral services for members of the enrolled families. The intention was to make use of all available local government and non-government resources to support the complete needs of families. The component included the trialling of “Patchwork”, a care-coordination digital web-based support tool developed in the UK by FutureGov [19].

**Evidence informed practice:** The overall design included evidence-informed elements: nurse-led sustained health home visiting, wrap-around care, family group conferencing, child and family service centres, and targeted and multimodal parenting programmes. Sustained nurse home visiting was implemented in parallel with the elements described here and provided an important new service for vulnerable families with infants less than 2 years of age. In addition to including evidence-informed elements in the design, the initiative included the establishment of Knowledge Translation Networks that would identify and promote the use of evidence-informed practice.

**General Practice engagement and support:** the engagement of families with a general practice, and supporting those general practices, was identified as a priority. The intention was to encourage families to have a general practice “health home”. An important objective of this component was to reduce emergency department and hospital admission for ambulatory and primary care sensitive conditions. The Australian Commonwealth funded Medical Benefits Scheme (MBS) is premised on the central coordinating role of family physicians in general practice. This component of the design sought to ensure that families had access to all available financial subsidies for their health and social care.

**Family Health Improvement:** The purpose of this component was to strengthen the delivery of public health and preventive health measures to families through the service network and general practice. Sector capacity building projects were proposed that would build on concurrent local initiatives including: a parenting communication initiative (“Love Talk Sing Read Play”) and a well childcare sector capacity building project. The intention was to also include preventive health measures into family care plans. Health protection measures for families were also identified as important. The design made provision for enhanced immunisation and healthy housing initiatives.

**Place-based initiatives:** The design assumed that for service integration to be successful it needed to be locally derived within a well organised primary and community sector. The design proposed two placed-based initiatives within the cities of Sydney and Canterbury-Bankstown. The design called for the trialling of wrap-around care in the place-based projects. The full nature of the local initiatives was not detailed in the design with intention of ensuring that they would be locally developed through community and consumer consultation.

**System Change:** The development of the integrated care design had occurred in the context of a strong cross agency collaborative partnership. The partnership was at that time developing a five-year Child Health and Wellbeing Plan and the integrated care initiative provided an opportunity to demonstrate many of the system change elements included in that plan. The integrated care initiative proposed that opportunities for shared planning, commissioning and evaluation be explored. A number of policies, tools and practices were identified including: informed consent to share information with partners, person-centred care, branding and promotion, web-based and social media tools, client and family self-assessment, robust and trusted privacy, and skilled and well-supported “health home” general practices. The proposal also sought to explore more significant system changes including: cross-agency “task group” models of care; funding and performance agreement changes to ensure shared outcomes in contracts; joint service delivery teams with shared accountability structures; and joined-up entities or new shared-purpose organisations.

**Child and family Outcomes:** The design identified the importance of having an outcome focus and included the monitoring of separate child and family outcomes. Included in this were several new initiatives that were considered central to integrated care, including: a shared outcome framework, patient reported outcome measures, patient reported experience measures, electronic medical record data-linkage projects, and population outcome monitoring.

**Evaluation:** The designed initiative was a “complex intervention” and consequently the evaluation framework drew on Medical Research Council guidance for evaluation of complex public health interventions [20,21,22]. As noted above, the overall research design is a longitudinal, multilevel, critical realist evaluation of applied programme interventions that seek to break intergenerational cycle of social disadvantage and poor child health and development outcomes. MRC guidance argues that only through close scrutiny of causal mechanisms is it possible for evaluation to contribute to developing more effective interventions, and provide insights into how findings might be transferred across settings and populations. Consequently a critical realist mixed method approach was chosen to examine the quantity and quality (or process) of what was actually implemented in practice, the context, the mechanisms and seek to answer the question why [13].

### Programme Theory

We have previously published the Programme Theory informing this design [3]. It is presented here to assist readers’ understanding of the design process.

### Theory of Change (ToC)

The overall design retained the four strategic themes of the local collaborative design process (i.e. improving system capacity; health and wellbeing promotion; early intervention and supporting place-based initiatives). The place-based initiatives, system change, and health and wellbeing strategies were proposed to be implemented using a co-design approach with local communities and interagency partners. Consequently detailed ToC logic models were not developed for components related to those interagency strategic themes.

**Figure 3:**
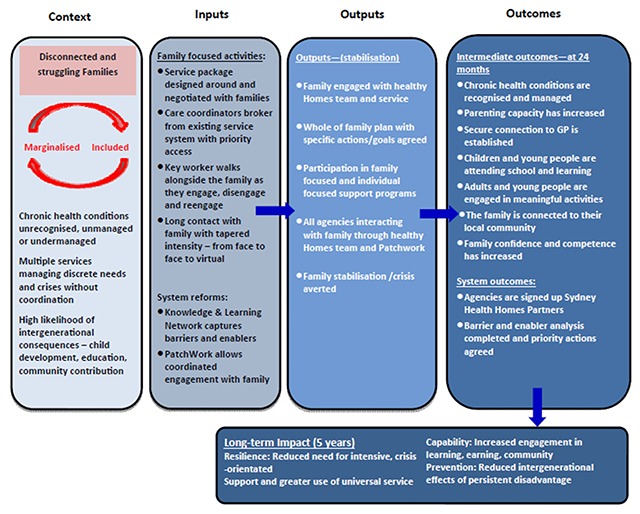
Theory of Change – Early Intervention and Clinical Elements.

By contrast the interagency partners sought to develop a ToC Logic Model that would guide the implementation of the early intervention and clinical aspects of the initiative. That ToC was particularly relevant for the 1) identification, 2) care coordination and 3) evidence-informed practice components of the design. It also sought to inform the clinical elements of the place-based projects. The ToC is shown at Figure 3.

**Figure 4:**
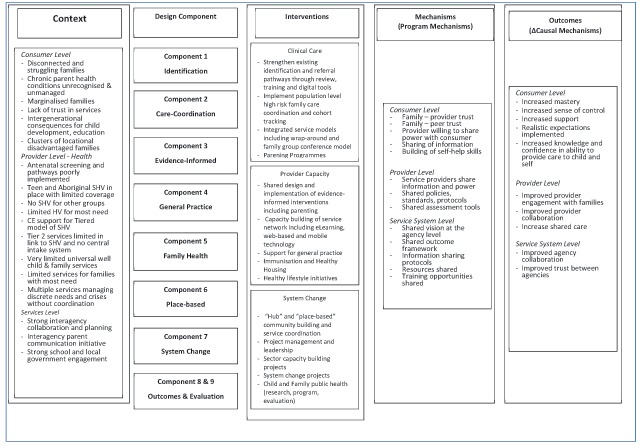
ToC Logic Model.

Prior to implementation in July 2015 a full Logic Model of the overall design was constructed to inform NSW Ministry of Health monitoring of the implementation. An adaption of that logic model is shown at Figure 4 with the inclusion of identified interventions and programme mechanisms.

## Discussion

In designing an integrated care initiative for vulnerable families we have drawn on our previously reported empirical studies, the translation of causal theory to programme theory, collaborative design processes, and the integrated care policy elements advanced by the NSW Ministry of Health. Critical realism has provided the methodological underpinning of this programme of work and has assisted to explicate both the contextual conditions and the underlying causal and programme mechanisms. Consequently we have been able to move from our earlier theoretical models toward the design of whole of health and social care system interventions.

In so doing we have moved from causal and programme mechanisms at the individual level toward mechanistic propositions relating to service systems and providers. For example, situated activity – face to face activity, and intermediate level social and service organisational mechanisms, continue to highlight the important of trust and willingness to share power. Thus the development and implementation of system change initiatives, such as general practice focused “health homes” and interagency “task groups” will be very reliant on approaches to sharing power and building trust between actors. To address those challenges the design has moved beyond the usual integrated care components of cohort identification, risk stratification and care-coordination to address the underlying programme mechanisms of trust and power sharing. Strong partnerships with general practice, and social and education sector partners, will be critical to the success of initiatives for children and families. True power sharing is difficult for health sector actors and will remain a challenge for the integrated care initiative described here.

The design seeks to address not only intergenerational cycles of violence, family dysfunction and psychopathology, but also the social determinants of health as described in our earlier empirical and theoretical work. Thus throughout the design attention has been given to: meeting all the material needs of families, improving access to services, reducing family social isolation and marginalisation, building local social networks and community cohesion, and improving community health and life-skill literacy.

Central to the initiative reported here is the strong interagency collaborative and the development of a shared outcomes framework. The final funding proposal included significant contributions from the local primary care agency, Family and Community Services NSW, non-government partners and academic institutions. A limitation was the lack of input from the Education sector which was attributable to restructuring of that sector at the time of design preparation. The consequence of this limitation is that the design has not fully explored the causal mechanisms operating within the school setting or the programme possibilities that might be operationalised through those settings. For example, the family-school dimension was not examined and consequently the potential of school initiated family support strategies are absent from the design.

A further limitation was the strong health sector focus despite the collective approach to planning. This can be attributed to the NSW Health integrated care tendering process, which was focused on initiatives for chronic health conditions. Despite this constraint, the final design was able to incorporate strong social care elements that took the design beyond the boundaries of the health sector. The underlying programme theory for the integrated care design remains tentative and will require further explication as part of the evaluation design.

The design propositions developed followed the context-intervention-mechanism-outcome (CIMO) logic advanced by Denyer [14]. That approach was extended beyond the theoretical to be included in the overall logic model. Thus the final logic model included both implementation theory and programme theory elements as proposed by Blamey and Mackenzie [18]. The value of considering and analysing the underlying causal, implementation, and programme mechanisms has been that consideration was given to questions of “why” and “how”. Consequently the design sought to build “mutually supporting” activities that would maximise the chances of success. Thus the analysis of how mechanisms operate in a context can help researchers to look out for, and establish potential areas of impact, through theorising about, and then establishing its causes. This can then facilitate the further refinement and improvement of programme design and implementation.

## Conclusion

The design study described advances our earlier empirical and programme design studies toward the implementation of a whole-of-government integrated health and social care initiative. That initiative was designed as a cross-agency care coordination network that would ensure that vulnerable families: had their complex health and social needs met; kept themselves and their children safe; and were connected to society. In so doing we aim to break intergenerational cycles of poverty, violence and crime, poor education and employment opportunities, psychopathology, and poor lifestyle and health behaviours, through strengthening family resiliency, improving access to services, and addressing the social determinants of health and wellbeing.
